# Errata

**Published:** 1882-12

**Authors:** 


### ERRATA.

Our readers are requested to note the following corrections, which should be made in the paper on "The value of graduated pressure in the treatment of diseases of the vagina, uterus, ovaries and other appendages," by Dr. Nathan Bozeman, which appeared in the December and January numbers of the REGISTER, Vol. II, Nos. 3 and 4. Unfortunately the proofs could not be sent to the author for revision, and the inevitable percentage of errors resulted:

On page 130, 8th line from bottom, read "with two" for "of two."
On page 135, 6th line from top, read "1869" for "1859."
On page 137, 7th line from top, read "Latin" for "English."
On page 137, 18th line from top, read "the New York Medical Journal."
On page 138, 3d line from top, read "as when" for "as."
On page 149, 11th line from top, read "fecal" for "fæcal."
On page 142, 20th line from top, read "the latter" for "it accurately."
On page 142, 20th line from top, read "pressure" for "hard pressure."
On page 149 and 151, read "Dr. Chauveau" for "Dr. Chanorin."
On page 151, 1st line from bottom, read "Fig. 3" for "Fig. 1"
On page 152, 15th line from top, read "depressor" for "depression."
On page 152, 17th line from top, read "balls" for "pads."
On page 154, 6th line from top, read "any form" for "my form."
On page 154, 17th line from top, read "form" for "former."
On page 155, 2nd line from top, read "there" for "these."
On page 200, 10th line from top, "the other" for "another."
On page 200, 13th line from bottom, read "the equivalent" for "as the acquivalent."
On page 205, 14th line from top, read "all" for "many cases."
On page 207 and others, read "Dr. Tauszky" for "Dr. Tanszky."
On page 214, 14th line from top, read "Dr. T.'s cut, Fig. 8."
On page 217, 7th line from top, read "Dr. T.'s second illustration."
On page 217, 3d line from bottom, read "pubes" for "pubis."
On page 226, 5th line from bottom, read "vesico-vaginal septum" for "anterior wall of the vagina in its normal condition."
On page 227, 3d line from top, read "fecal" for "local."
On page 227, 25th line from top, read "sponge columning" for "the cotton."
On page 29 4th line from top, expunge "the most," and read "little" for "the east."

